# Early left ventricular microvascular dysfunction in diabetic pigs: a longitudinal quantitative myocardial perfusion CMR study

**DOI:** 10.1186/s12933-023-02106-w

**Published:** 2024-01-06

**Authors:** Li Jiang, Wei‑Feng Yan, Lu Zhang, Hua‑Yan Xu, Ying‑Kun Guo, Zhen-Lin Li, Ke-Ling Liu, Ling-Ming Zeng, Yuan Li, Zhi-Gang Yang

**Affiliations:** 1 Department of Radiology, West China Hospital, Sichuan University, 37# Guo Xue Xiang, Chengdu, Sichuan 610041 China; 2 Department of Radiology, Key Laboratory of Birth Defects and Related Diseases of Women and Children of Ministry of Education, West China Second University Hospital, Sichuan University, 20# South Renmin Road, Chengdu, Sichuan 610041 China

**Keywords:** Microvascular dysfunction, Diabetes, Quantitative myocardial perfusion, Myocardial microvascular reserve function.

## Abstract

**Background:**

Microvascular pathology is one of the main characteristics of diabetic cardiomyopathy; however, the early longitudinal course of diabetic microvascular dysfunction remains uncertain. This study aimed to investigate the early dynamic changes in left ventricular (LV) microvascular function in diabetic pig model using the cardiac magnetic resonance (CMR)-derived quantitative perfusion technique.

**Methods:**

Twelve pigs with streptozotocin-induced diabetes mellitus (DM) were included in this study, and longitudinal CMR scanning was performed before and 2, 6, 10, and 16 months after diabetic modeling. CMR-derived semiquantitative parameters (upslope, maximal signal intensity, perfusion index, and myocardial perfusion reserve index [MPRI]) and fully quantitative perfusion parameters (myocardial blood flow [MBF] and myocardial perfusion reserve [MPR]) were analyzed to evaluate longitudinal changes in LV myocardial microvascular function. Pearson correlation was used to analyze the relationship between LV structure and function and myocardial perfusion function.

**Results:**

With the progression of DM duration, the upslope at rest showed a gradually increasing trend (P = 0.029); however, the upslope at stress and MBF did not change significantly (P > 0.05). Regarding perfusion reserve function, both MPRI and MPR showed a decreasing trend with the progression of disease duration (MPRI, P = 0.001; MPR, P = 0.042), with high consistency (r = 0.551, P < 0.001). Furthermore, LV MPR is moderately associated with LV longitudinal strain (r = − 0.353, P = 0.022), LV remodeling index (r = − 0.312, P = 0.033), fasting blood glucose (r = − 0.313, P = 0.043), and HbA1c (r = − 0.309, P = 0.046). Microscopically, pathological results showed that collagen volume fraction increased gradually, whereas no significant decrease in microvascular density was observed with the progression of DM duration.

**Conclusions:**

Myocardial microvascular reserve function decreased gradually in the early stage of DM, which is related to both structural (but not reduced microvascular density) and functional abnormalities of microvessels, and is associated with increased blood glucose, reduced LV deformation, and myocardial remodeling.

**Supplementary Information:**

The online version contains supplementary material available at 10.1186/s12933-023-02106-w.

## Background

Diabetes mellitus (DM) has become a major public health problem worldwide because of its high prevalence and rapid growth [1]. Because of its microvascular and macrovascular complications, DM is one of the major causes of mortality and morbidity, particularly premature death [1, 2]. Studies have indicated that microvascular pathology is one of the main characteristics of diabetic cardiomyopathy; however, most studies were cross-sectional studies, and the longitudinal course of microvascular disorder in DM remains uncertain [3–5]. Moreover, because microvascular complications are often silent in their early stages, they can be nearly incurable once they appear [2]. Thus, elucidating the process of microvascular dysfunction, identifying individuals at a high risk of microvascular complications at an early stage, and guiding therapeutic options to prevent the development and progression of these complications are strongly needed.

Cardiac magnetic resonance (CMR) has become an important tool in myocardial injury evaluation and longitudinal follow-up cardiac examination because of its advantages of being radiation-free, multi-sequence, and multi-parameter and high repeatability [6–8]. CMR first-pass contrast-enhanced perfusion imaging has evolved into an established technique with a good clinical application over the past 2 decades [5, 9–11]. With the development of the technology, CMR permits the quantitative measurement of the myocardial blood flow (MBF) and myocardial perfusion reserve (MPR) in vivo and has been well validated, which provides a feasible technical means for longitudinal and quantitative evaluation of the occurrence and development of early microvascular disorders related to DM [12–14].

Because animal models can provide necessary pathological validation and convenient and controllable longitudinal observation, they serve as powerful tools for studying disease mechanisms and the development of DM-related heart diseases. Pigs are attractive animal models because of their similarities to humans in anatomy and metabolism and can bridge the gap between basic studies and clinical trials in human patients [15].

Therefore, this study aimed to investigate the early dynamic changes in left ventricular (LV) microvascular function in a pig model of DM using the CMR-derived quantitative perfusion technique, to analyze the relationship between LV microvascular function and structure and strain, to verify the feasibility of the fully quantitative perfusion technique in early evaluation of microvascular function, and to provide imaging evidence for guiding the implementation of new therapeutic options for preventing the development and progression of microvascular complications of DM.

## Methods

### Animals

These animal experiments complied with the Animal Research: Reporting of In Vivo Experiments guidelines and have been approved by our hospital’s Animal Ethics Committee (No.2,019,149 A).

This was a self-controlled longitudinal study involving 16-week-old female Bama minipigs. The included subjects were pigs with DM successfully induced using streptozotocin (STZ). The criterion for the success of an STZ-induced DM model was that the fasting blood glucose (FBG) of experimental pigs continued to exceed 7 mmol/L 1 month after the injection of STZ. To reduce the damage caused by extreme hyperglycemia, insulin is administered in moderation to ensure that the FBG of pigs with DM is < 20 mmol/L. The diabetic modeling process and feeding methods have been previously described in detail [16].

**Fig. 1:**
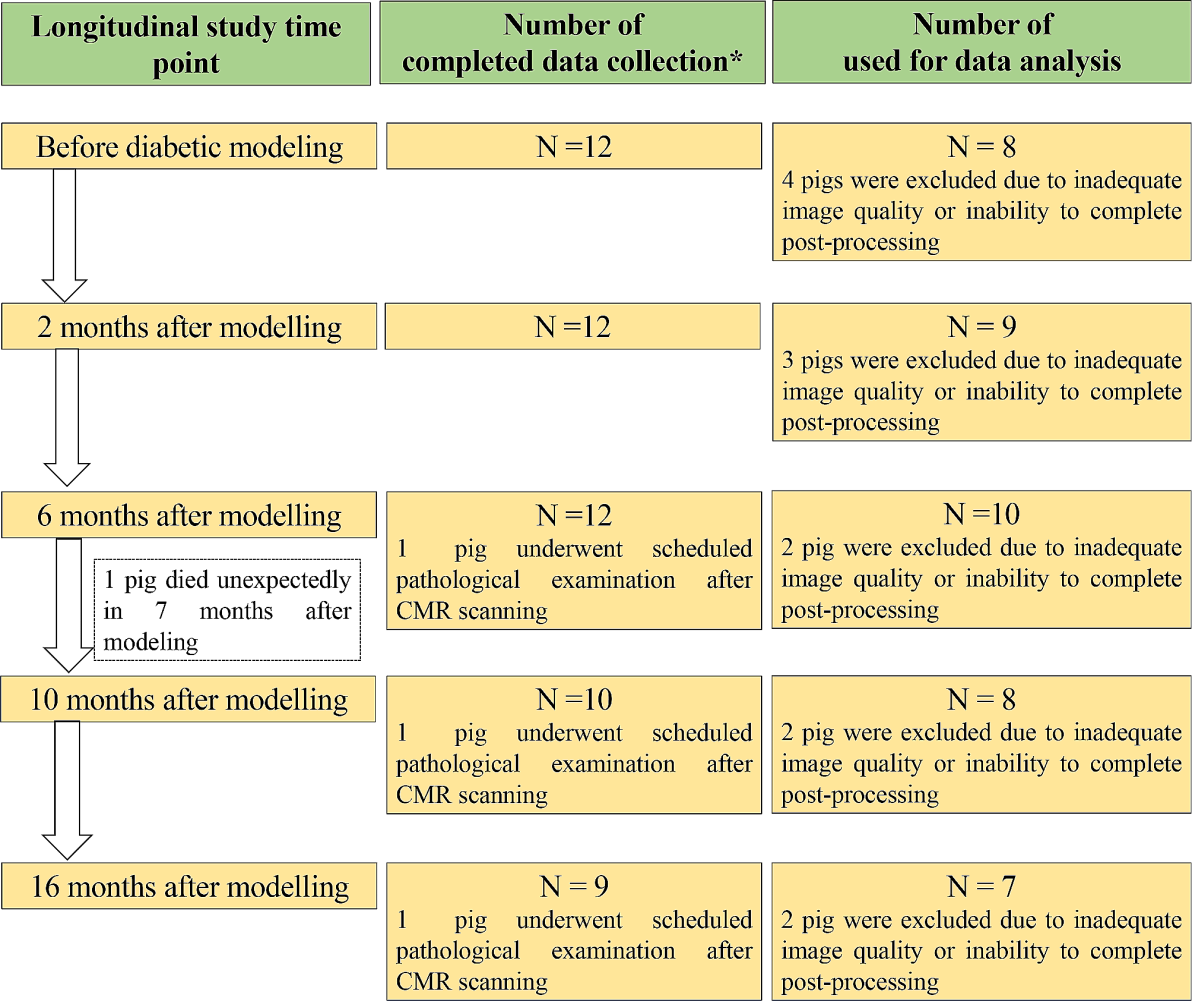
Flow chart of the experimental procedure

Longitudinal observation of the pigs included CMR scanning, blood biochemistry, and histological examination (if necessary) before and 2, 6, 10, and 16 months after diabetic modeling (Fig. 1).

### CMR protocol

A 3.0 T whole-body MR scanner (Magneton Skyra, Siemens Medical Solutions, Erlangen, Germany) was used to perform longitudinal CMR scans. To ensure CMR image quality, the animals were intubated before scanning and connected to the animal anesthesia ventilator to maintain anesthesia and control breathing (respiratory rate, 10–30 respirations/min; inhalation/breathing ratio, 1:2). During the scanning process, breath holding was performed if necessary. A standard echocardiography (ECG)-triggering device was used to monitor ECG dynamic changes during image acquisition. The balanced steady-state free precession sequence (repetition time (TR)/echo time (TE), 3.15/1.36 ms; flip angle, 35°; slice thickness, 6.5 mm; matrix, 154 × 192 pixels, and field of view, 400 × 320 mm^2^) was performed covering the LV on the short-axis view for continuous cine imaging and two-, three-, and four-chamber cine imaging on the long-axis view.

CMR first-pass contrast-enhanced perfusion images were acquired at stress and rest. Stress perfusion images were acquired following the administration of intravenous adenosine (Solarbio, China) at 140 µg/kg/min for 5 min. A dual-bolus contrast agent scheme was used to correct for signal saturation of the arterial input function (AIF). Briefly, 0.01-mmol/kg gadopentetate dimeglumine (Magnevist, Bayer HealthCare Pharmaceuticals, Wayne, New Jersey) was administered as a pre-bolus. Immediately, first-pass perfusion data were acquired after injecting 0.1-mmol/kg gadolinium at 2 mL/s, followed by 20-mL 0.9% saline. Resting perfusion imaging was performed after a minimum of 15 min following stress acquisition according to the Society for Cardiovascular Magnetic Resonance guidelines [17–19]. First-pass perfusion was acquired concurrent with intravenous contrast agents in three standard short-axis slices (the basal, mid-ventricular, and apical slices) and performed by inversion recovery prepared echoplanar sequence (TR/TE, 148/1.1 ms; flip angle, 35°; matrix, 154 × 192 pixels, and field of view, 258 × 322 mm^2^).

### Image analysis

All images were transferred to cvi42 (Circle Cardiovascular Imaging Inc.), offline commercial software, for postprocessing analysis. According to the current postprocessing guidelines, the LV morphological (myocardial mass, end-diastolic volume [EDV], end-systolic volume [ESV], and remodeling index [Calculated as LV myocardial mass/EDV]), functional (ejection fraction [EF]), and strain (longitudinal global peak strain [GPS-L]) parameters were measured based on the analysis of continuous short-axis and two-, three-, and four-chamber long-axis cine images [20].

**Fig. 2:**
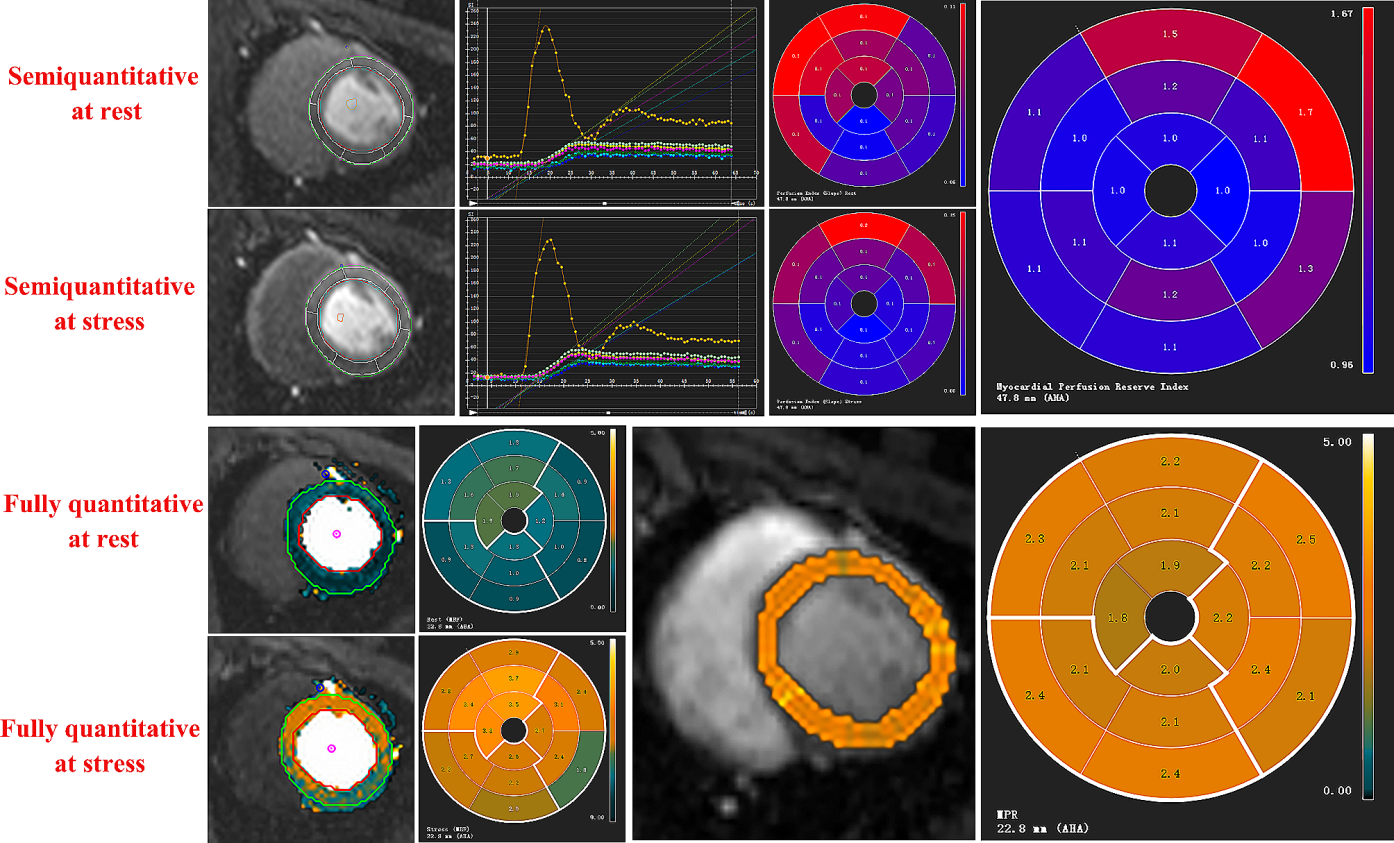
CMR-derived semiquantitative and fully quantitative perfusion analysis

CMR-derived semiquantitative and fully quantitative perfusion analyses were performed using signal-intensity curves during the first-pass of gadolinium [21]. For semiquantitative perfusion analysis, the stress and rest myocardial perfusion images were imported into the corresponding functional regions, and semiquantitative perfusion parameters, including maximal signal intensity (MaxSI, derived from signal-intensity curves), upslope (maximum signal intensity increase over time), perfusion index (PI, calculated as upslope[myocardium]/Upslope [blood pool]), and myocardial perfusion reserve index (MPRI, representing the ratio of PI [stress]/PI [rest]), were automatically generated after accurate identification of the endo- and epicardium and blood pools. For fully quantitative perfusion analysis, the AIF and myocardial perfusion images at stress and rest were imported into corresponding software modules for analysis, and fully quantitative parameters, including MBF at rest, MBF at stress, and MPR (representing the ratio of stress MBF/rest MBF), were automatically generated, and manually adjusted when appropriate. The autogenerated perfusion parameters were represented using a 16-segment model (Bull’s eye plot) and three standard short-axis slices (basal, mid-ventricular, and apical slices). The global perfusion parameters were calculated as the average parameter value of the 16 segments in this study (Fig. 2).

### Histology and immunohistochemistry

Following imaging scans, 6, 10, and 16 months after the successful modeling, three pigs were randomly selected from each stage to be euthanized using 20-mL potassium chloride under deep anesthesia to obtain immediate pathological tissue. After cardiac arrest, the heart was removed immediately, flushed with saline solution, and then cut into 5-mm slices. Then, the slices were prefixed, dehydrated, paraffin-embedded, and cut into 5-µm sections for microscopic examination of the myocardium. For histopathological analysis, sections were stained with Masson’s trichrome stain to observe changes in myocardial tissue deposition. For immunohistochemical analysis, CD31 monoclonal antibody (Abcam, ab281583) was used to label microvascular vascular endothelial cells in the myocardium to measure microvascular density. The microvascular density was counted using Image-Pro Plus 6.0 software (Media Cybernetics). Take 3 fields of view (magnification, × 400) in a sample to obtain the average.

### Intra- and interobserver reproducibility

The intra- and interobserver variability for LV myocardial perfusion parameters was analyzed in 15 randomly selected original CMR images. To determine the intraobserver variability, an investigator evaluated the same images in two sessions at a 1-month interval. For the interobserver variability, a second investigator was blinded to the first investigator’s results and clinical data were reanalyzed.

### Statistical analysis

Statistical analyses were performed using Statistical Package for the Social Sciences (version 24.0; IBM, Armonk, NY, USA). Continuous data are expressed as the means ± standard deviations. The baseline characteristics and CMR parameters (LV geometric and functional, semiquantitative myocardial perfusion, and fully quantitative myocardial perfusion parameters) in experimental animals with the progression of disease duration were compared at different DM durations using linear mixed model. Pearson correlation was used to analyze the correlation between fully quantitative myocardial perfusion (MBF at rest, MBF at stress, and MPR) and LV MPRI, strain, LV EF, LV remodeling index, FBG, and glycosylated hemoglobin (HbA1c). The intraclass correlation coefficient (ICC) was used to measure the variability of CMR myocardial perfusion parameters. A two-tailed P-values < 0.05 were used to denote statistical significance.

## Results

### Baseline characteristics of the experimental animals

Twelve pigs with STZ-induced DM were included in this study for longitudinal analysis. During the follow-up, one pig died unexpectedly 7 months after modeling for unknown reasons, and three pigs underwent scheduled pathological examination 6, 10, and 16 months after modeling. The final number of animals included in this study for analysis is shown in Fig. 1. The baseline characteristics of the animals under study are shown in Table 1. After modeling, FBG (> 12 mmol/L) and HbA1c were significantly increased, which confirmed the successful modeling of DM. Similarly, urea and creatine kinase-MB (CK-MB) were significantly increased after DM modeling.

**Table 1:** Baseline characteristics of experimental animals

	Before modelling (n = 8)	2 months after modelling (n = 9)	6 months after modelling (n = 10)	10 months after Modelling (n = 8)	16 months after modelling (n = 7)	P value
Age (months)	16	18	22	26	32	-
Weight (kg)	23.44 ± 5.45	30.18 ± 6.55	36.00 ± 10.59*	42.31 ± 11.74*§	42.36 ± 9.82*§	0.001
FBG (mmol/l)	3.60 ± 0.59	13.15 ± 4.23*	13.36 ± 5.48*	18.10 ± 5.52*§‡	15.20 ± 2.18*	< 0.001
HbA1c (%)	3.01 ± 0.36	3.37 ± 1.02	5.60 ± 1.31*§	4.95 ± 1.45*§	6.08 ± 0.78*§	< 0.001
TC (mmol/l)	5.34 ± 6.09	2.64 ± 0.80	3.73 ± 3.58	3.77 ± 4.81	2.11 ± 0.59	0.258
HDL (mmol/l)	1.14 ± 0.65	0.76 ± 0.36	0.53 ± 0.42	1.08 ± 1.81	0.54 ± 0.24	0.156
LDL (mmol/l)	2.68 ± 3.03	7.24 ± 8.55*	1.98 ± 1.95§	1.85 ± 1.84§	1.27 ± 0.40§	0.215
Creatinine (µmol/l)	70.50 ± 25.08	64.38 ± 14.20	66.20 ± 22.18	39.00 ± 3.61*	51.28 ± 15.75	0.001
Urea (µmol/l)	2.65 ± 1.02	4.30 ± 1.13*	4.94 ± 0.83*	6.01 ± 0.94*§‡	5.40 ± 1.27*	< 0.001
CK-MB (µg/l)	0.37 ± 0.29	0.63 ± 0.73	3.15 ± 2.34*§	3.62 ± 3.17*§	2.83 ± 2.38*§	0.004

*Note*: Data expressed as means ± standard deviation (± SD)

FBG, fasting blood glucose; HbA1c, glycosylated hemoglobin; TC, triglyceride; HDL, high density lipoprotein cholesterol; LDL, low-density lipoprotein cholesterol; CK-MB, Creatine kinase MB

* *P* < 0.05 versus before modelling

§ *P* < 0.05 versus 2 months after modelling

‡*P* < 0.05 versus 6 months after modelling

# *P* < 0.05 versus 10 months after modelling

### Longitudinal changes in LV geometry and strain in pigs with DM

The CMR-derived LV morphological, functional, and strain parameters in the experimental animals are shown in Table 2. With the progression of disease duration, no significant reduction in LV EF was observed (P = 0.420). However, the LV EDV and ESV first gradually increased and decreased at the 10th month after modeling (P = 0.024 and 0.006, respectively). Similarly, the LV remodeling index first gradually decreased and increased at the 10th month after modeling (P = 0.002). Nevertheless, the LV GPS-L showed a gradual decrease with the progression of disease duration, and the decrease was most obvious at the 16th month after modeling (− 10.15% ± 1.68% *vs. −*9.53% ± 2.38% vs. −9.36% ± 2.97% vs. −8.31% ± 2.09% vs. −6.51% ± 2.22%; P = 0.039).

**Table 2:** LV structure and function in diabetic pigs with the progression of diabetes duration

	Before modelling (n = 8)	2 months after modelling (n = 9)	6 months after modelling (n = 10)	10 months after Modelling (n = 8)	16 months after modelling (n = 7)	P value
**LV Geometric and functional**						
EDV (ml)	34.25 ± 11.50	35.89 ± 8.16	43.62 ± 6.57*	33.07 ± 7.05‡	28.14 ± 11.72‡	0.024
ESV (ml)	15.39 ± 6.86	14.66 ± 5.52	16.57 ± 8.74	10.34 ± 4.45	7.56 ± 3.03*§‡	0.006
SV (ml)	18.86 ± 5.87	21.22 ± 6.69	27.06 ± 5.68*§	22.73 ± 5.44	20.57 ± 9.72	0.099
EF (%)	55.01 ± 7.36	54.63 ± 11.33	57.57 ± 11.52	61.21 ± 9.99	63.08 ± 11.61	0.420
Heart rate	93 ± 14	86 ± 15	105 ± 23	98 ± 21	87 ± 16	0.248
Mass (g)	38.16 ± 6.41	41.80 ± 10.14	50.00 ± 14.09	49.18 ± 7.43*	53.59 ± 8.65*§	0.009
Remodeling index (g/ml)	1.43 ± 0.36	1.16 ± 0.29	1.14 ± 0.39	1.45 ± 0.39	2.22 ± 1.11*§‡#	0.002
**LV Strain**						
GPS-L (%)	−10.15 ± 1.68	−9.53 ± 2.38	−9.36 ± 2.97	−8.31 ± 2.09	−6.51 ± 2.22*§‡	0.039

*Note*: Data expressed as means ± standard deviation (± SD)

EDV, End-diastolic volume; ESV, End-systolic volume; SV, Stroke volume; EF, Ejection fraction; GPS-L, Longitudinal global peak strain

* *P* < 0.05 versus before modelling

§ *P* < 0.05 versus 2 months after modelling

‡*P* < 0.05 versus 6 months after modelling

# *P* < 0.05 versus 10 months after modelling

### Longitudinal changes in myocardial perfusion function using the semiquantitative perfusion analysis technique

The semiquantitative myocardial perfusion parameters, such as upslope, MaxSI, PI, and MPRI, in the animals under study are shown in Table 3. With the progression of disease duration, the LV upslope at rest showed a gradually increasing trend (P = 0.029), while the upslope at stress did not change significantly (P = 0.377), and it was always higher at stress than at rest. No obvious regular changes in PI at different durations of DM were observed (P = 0.947 at rest; P = 0.743 at stress). However, for MPRI, a significant decreasing trend was observed with the progression of disease duration, and the decrease was most obvious at the 16th month after modeling (1.16 ± 0.18 vs. 1.13 ± 0.22 vs. 1.09 ± 0.16 vs. 1.00 ± 0.17 vs. 0.86 ± 0.09; P = 0.001).

**Table 3:** CMR-derived semi-quantitative and fully quantitative myocardial perfusion analysis

	Before modelling (n = 8)	2 months after modelling (n = 9)	6 months after modelling (n = 10)	10 months after Modelling (n = 8)	16 months after modelling (n = 7)	P value
**Semi-quantitative myocardial perfusion**						
Upslope at rest (SI/sec)	2.27 ± 0.86	2.64 ± 0.73	3.22 ± 1.67	3.12 ± 1.41	3.84 ± 0.88*§	0.029
Upslope at stress (SI/sec)	3.25 ± 1.05	3.25 ± 1.37	3.89 ± 2.36	3.91 ± 1.65	4.40 ± 1.31	0.377
MaxSI at rest (SI)	37.90 ± 6.94	38.16 ± 8.47	36.25 ± 11.56	40.76 ± 13.53	47.28 ± 6.66‡	0.074
MaxSI at stress (SI)	36.63 ± 6.34	37.81 ± 9.42	32.75 ± 10.52	40.15 ± 15.08	44.43 ± 5.76‡	0.061
PI at rest (%)	9.67 ± 3.40	10.16 ± 1.85	10.19 ± 3.80	12.47 ± 10.10	10.56 ± 2.28	0.947
PI at stress (%)	10.58 ± 2.79	10.92 ± 2.25	11.58 ± 5.43	12.66 ± 9.89	9.7 ± 2.27	0.743
MPRI	1.16 ± 0.18	1.13 ± 0.22	1.09 ± 0.16	1.00 ± 0.17	0.86 ± 0.09*§‡	0.001
**Fully quantitative myocardial perfusion**						
MBF at rest (mL/g/min)	1.32 ± 0.37	1.52 ± 0.80	1.56 ± 0.58	1.55 ± 0.65	1.92 ± 0.63	0.322
MBF at stress (mL/g/min)	1.88 ± 0.48	2.18 ± 0.92	2.24 ± 1.00	2.26 ± 0.97	2.07 ± 0.69	0.760
MPR	1.84 ± 0.29	1.75 ± 0.40	1.58 ± 0.30	1.57 ± 0.28	1.24 ± 0.40*§	0.042

*Note*: Data expressed as means ± standard deviation (± SD)

MaxSI, maximal signal intensity; PI, perfusion index, MPRI, myocardial perfusion reserve index; MBF, myocardial blood flow; MPR, myocardial perfusion reserve

* *P* < 0.05 versus before modelling

§ *P* < 0.05 versus 2 months after modelling

‡*P* < 0.05 versus 6 months after modelling

# *P* < 0.05 versus 10 months after modelling

### Longitudinal changes in myocardial perfusion function using the fully quantitative perfusion analysis technique

**Fig. 3:**
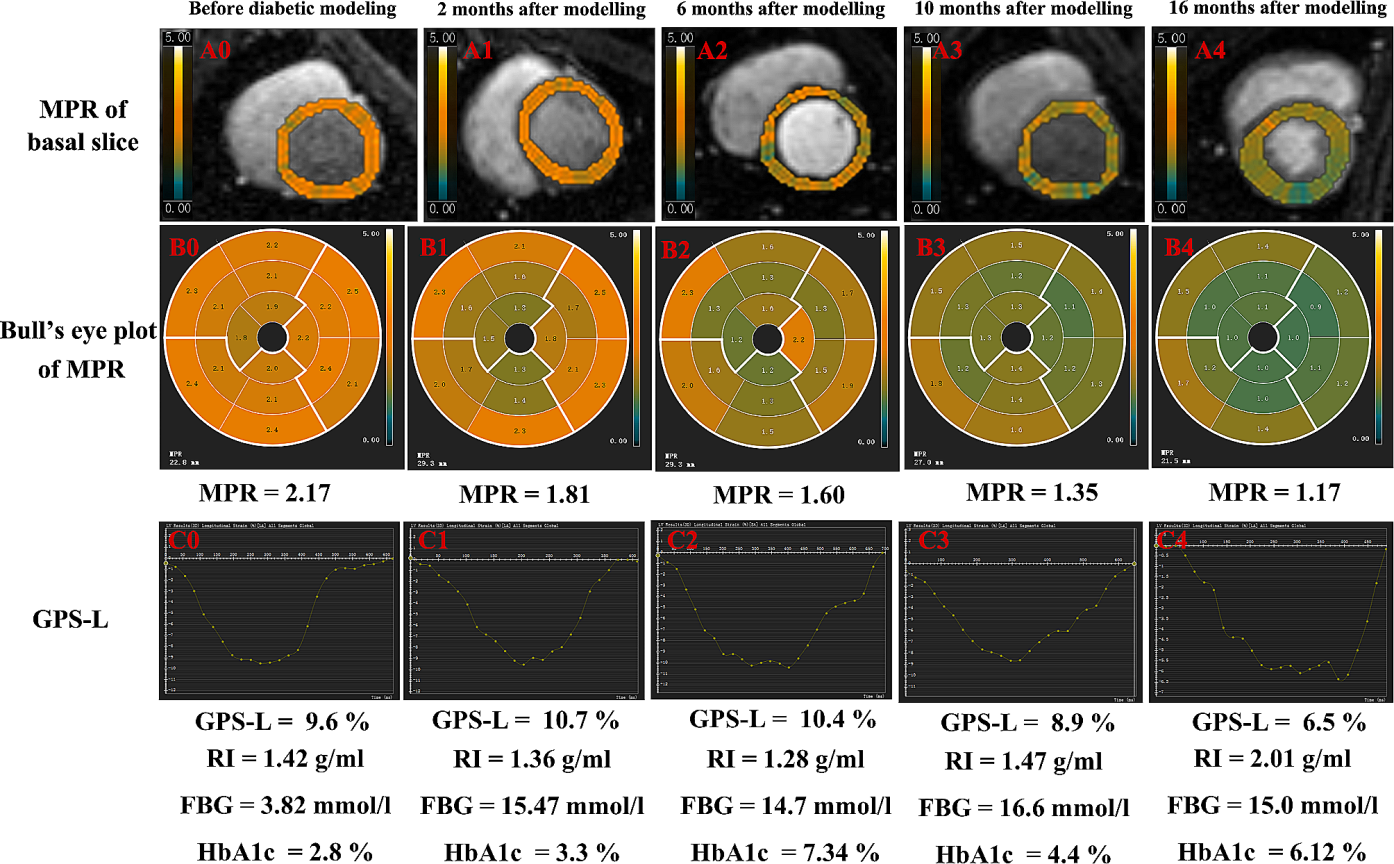
Representative case shows the time course of LV MPR, morphology, function, and some metabolic parameters. This case shows that the LV MPR and GPS-L decrease gradually with the progression of DM duration. RI, Remodeling index; Other abbreviations are listed in Tables 1 and 3

The fully quantitative myocardial perfusion parameters, such as MBF and MPR, in the animals under study are shown in Tables 3 and Fig. 3. With the progression of disease duration, the MBF at rest and stress did not change significantly (P = 0.322 at rest; P = 0.760 at stress). As for the MPR, consistent with the trend of the MPRI obtained using the semiquantitative perfusion technique, it also showed a significant decreasing trend (1.84 ± 0.29 *vs*. 1.75 ± 0.40 *vs*. 1.58 ± 0.30 *vs*. 1.57 ± 0.28 *vs*. 1.24 ± 0.40; P = 0.042).

### Correlations between myocardial perfusion and other relevant variables

**Fig. 4:**
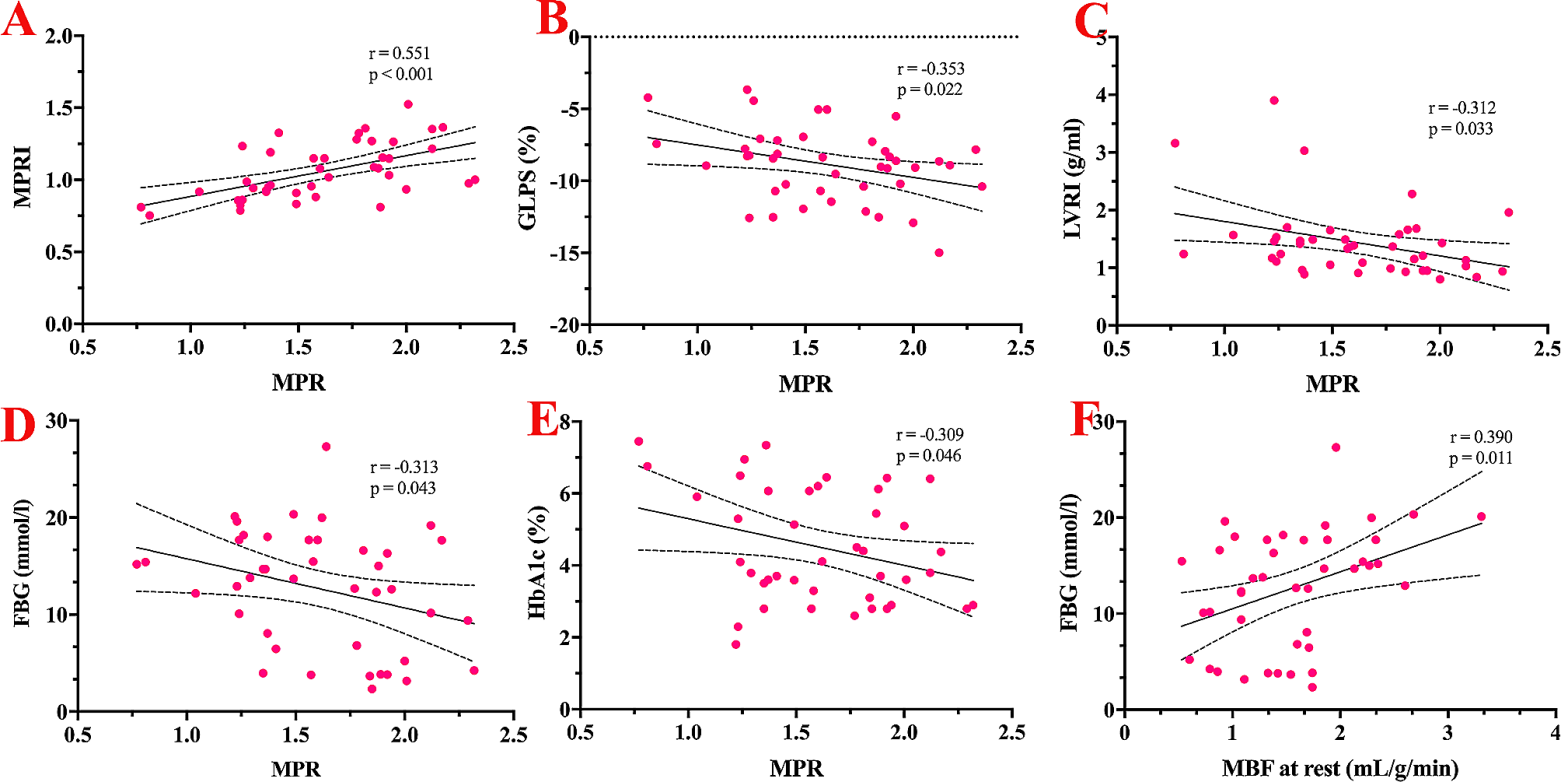
Correlations between myocardial perfusion and LV strain and LVRI. The LV MPR was significantly correlated with the MPRI and moderately associated with the LV GPS-L, LV remodeling index, FBG, and HbA1c. The abbreviations are listed in Tables 1 and 3

As shown in Tables 4 and Fig. 4, the LV MPR (fully quantitative parameter) was significantly correlated with the MPRI (semiquantitative parameter) (r = 0.551, P < 0.001) and moderately associated with the LV GPS-L (r = − 0.353, P = 0.022), LV remodeling index (r = − 0.312, P = 0.033), FBG (r = − 0.313, P = 0.043), and HbA1c (r = − 0.309, P = 0.046). The LV MBF at rest was moderately associated with the LV GPS-L (r = 0.373, P = 0.015). However, the MBF at stress was only moderately associated with FBG (r = 0.384, P = 0.012).

**Table 4:** Correlation between fully quantitative perfusion and semi-quantitative perfusion, strain and remodeling index

	MBF at rest			MBF at stress			MPR	
	r	p		r	p		r	p
MPRI	−0.235	**0.045**		0.045	0.778		0.551	**< 0.001**
GPS-L (%)	0.373	**0.015**		0.231	0.142		−0.353	**0.022**
EF (%)	0.020	0.899		−0.227	0.148		−0.238	0.129
Remodeling index (g/ml)	0.089	0.573		−0.188	0.234		−0.312	**0.033**
FBG (mmol/l)	0.390	**0.011**		0.384	**0.012**		−0.313	**0.043**
HbA1c (%)	0.217	0.168		0.169	0.284		−0.309	**0.046**

Bold values indicate statistical significance (P-values < 0.05). The abbreviations are listed in Tables 1 and 3

### Histological and immunohistochemical analyses

**Fig. 5:**
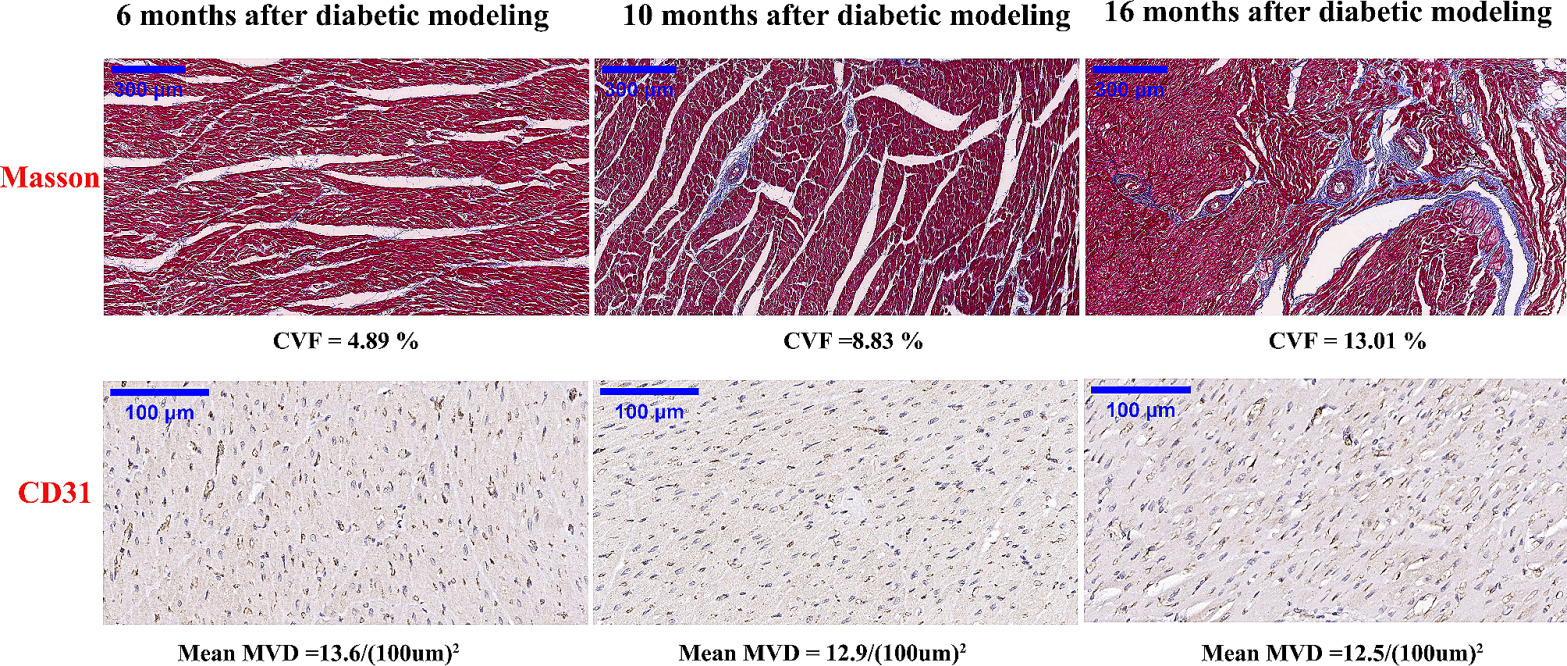
Masson’s trichrome and CD31 staining of the myocardium in diabetic pigs with different disease courses. Masson staining showed that fibrosis was deposited in the extracellular space and surrounding blood vessels of the myocardium, and CVF increased gradually; CD31 staining showed no significant decrease in MVD (< 10%) with the progression of disease duration. CVF, collagen volume fraction; MVD, microvascular density

Microscopically, Masson’s trichrome and CD31 staining exhibited myocardial fibrosis and microvessels in the myocardium at different modeling durations. As shown in Fig. 5, Masson’s trichrome staining showed that fibrosis was deposited in the extracellular space and surrounding blood vessels of the myocardium, and collagen volume fraction increased gradually with the progression of disease duration. CD31 staining showed no significant decrease in microvascular density (< 10%), which was inconsistent with the MPR trend.

### Reproducibility of myocardial perfusion measurement

The intra- and interobserver correlation variability analysis is summarized in additional file: Table S1. The intra- and interobserver agreements were considered good in measuring LV semiquantitative myocardial perfusion (ICC = 0.929–0.989 and 0.924–0.972, respectively) and LV fully quantitative myocardial perfusion (ICC = 0.961–0.987 and 0.925–0.975, respectively).

## Discussion

In this study, we established STZ-induced DM models to longitudinally analyzed early dynamic changes in LV myocardial microvascular function using the semiquantitative and fully quantitative perfusion techniques generated by CMR first-pass perfusion images, yielding several interesting findings, as follows: (1) both semiquantitative and fully quantitative myocardial perfusion results indicated that the myocardial microvascular reserve function of diabetic pigs gradually decreased with the progression of disease duration, and the results obtained using the two techniques were highly consistent; (2) reduced myocardial microvascular reserve function is associated with increased blood glucose levels, reduced LV deformation, and myocardial remodeling; (3) the increase in microcirculation perivascular fibrosis was consistent with the gradual decrease in myocardial microvascular reserve function; however, microvascular density did not significantly change in the early stages of DM.

Increasing evidence has demonstrated the importance of coronary microvascular dysfunction and its apparent correlation with prognosis, and CMR first-pass perfusion imaging is considered an important method for evaluating myocardial microvascular function [22, 23]. In our study, both the semiquantitative and fully quantitative perfusion techniques were used to evaluate the longitudinal changes in myocardial microcirculation with the progression of DM duration, and the myocardial perfusion reserve function obtained using the two techniques was highly consistent, which confirmed the feasibility of the two techniques and indicated that our results are more reliable.

The myocardial microvascular reserve function has classically been considered the gold standard for examining microvascular function [24]. In the absence of obstructive coronary artery disease, impaired MPR has been shown to be able to predict the occurrence of adverse cardiovascular events and an independent predictor of cardiac mortality [25]. In this sequential longitudinal study, both semiquantitative and fully quantitative myocardial perfusion analyses indicated that the myocardial microvascular reserve function of diabetic pigs gradually decreased with the progression of DM duration, which is consistent with the findings of previous studies suggesting that diabetes can cause microcirculation damage [26], and we furthermore verified the early and gradually worsening myocardial microvascular function caused by DM, suggesting the importance of early treatment. Numerous studies have indicated that both microvascular functional and structural injuries may contribute to microvascular dysfunction [27], and the structural injuries may be associated with microvascular rarefaction, perivascular fibrosis, and limited vasodilation because of myocardial remodeling [28]. This study has shown degree of myocardial fibrosis increased gradually with the progression of disease duration, and the myocardial remodeling was obvious in the later stage of follow-up; however, we did not find any reduction in the density of microvessels, suggesting that microvascular rarefaction occurs later in the course of DM.

According to our data and previous findings, we believe that reduced MPR is caused by increased resting MBF and the inability of MBF to increase proportionately with resting MBF during stress [24]. In DM, the myocardium relies heavily on the metabolism of fatty acids for energy. However, fatty acid oxidation requires more oxygen than glucose oxidation, resulting in a higher basal oxygen requirement and subsequently a higher resting MBF [24]. With the progression of DM duration, because of the intrinsic sensitivity of β-oxidation to ischemia and because the excessive accumulation of reactive oxygen species reduces the capacity of the myocardium to oxidize fatty acids during β-oxidative metabolism, ultimately, the basal oxygen requirement continues to increase [29]. In this study, although the MBF at rest showed a gradually increasing trend, there was no statistical difference. We speculated that the small sample size and short follow-up time limited the statistical difference. Thus, larger samples with a longer follow-up are warranted to validate these findings. Furthermore, our study found that FBG is positively correlated with the MBF and negatively correlated with MPR, suggesting that blood glucose levels can affect MBF by regulating metabolism and thus contribute to the disease process [30, 31].

Similar to the trend described in our previous study [16], the LV longitudinal strain and remodeling index changed significantly in the later stage of follow-up (16 months after modeling) and were correlated with MPR. Because the LV GPS-L, LV remodeling index, and MPR had no significant changes in the early stage, but showed a trend of gradual deterioration, the time sequence of injury/dysfunction was approximated, and no obvious causal relationship was found. Considering the DM-related injury pathways and mechanisms, combined with the longitudinal CMR results and related pathological verifications in this study, it is concluded that DM-related multi-mechanisms/pathways work together to simultaneously cause damage to the heart structure, function, and microcirculation and interfere with each other through metabolic changes, energy deprivation, structural restriction, and other pathways. These results suggest that early intervention is the key to breaking a chain reaction.

### Limitation

Our study also had several limitations. First, to ensure long-term longitudinal follow-up of the models, only three animals were selected for pathological examination, and we will increase pathological samples to verify our results in the future. Second, myocardial damage in DM is a long-term chronic process. In this study, we only conducted a longitudinal assessment of myocardial microvascular dysfunction in the early stage of DM, which may be the reason why no significant changes in microvascular density were observed in the study, and the relevant studies on myocardial damage in the later stage of DM will be illustrated in a subsequent study. Third, considering that the body weight of uncontrolled diabetic pigs was significantly lower than that of the control group at the same age [32], we used a self-controlled longitudinal study to reduce the interference of confounding factors. Longitudinal cohort studies free of weight interference will attempt to address this issue in future research.

## Conclusions

In this study, CMR-derived semiquantitative and fully quantitative perfusion techniques highlighted myocardial microvascular function decreased gradually in the early stage of DM using large animal models, and demonstrated that reduced myocardial microvascular function is associated with increased blood glucose, reduced LV deformation, and myocardial remodeling. Furthermore, combined with pathological results, it indicated both structural and functional abnormalities of microvessels contributed to the early reduction in myocardial microvascular function. In summary, the progressive deterioration of myocardial microvascular function in the early stages of DM, demonstrated in this study, suggests the importance of early intervention in DM-related microvascular disorders and provides a feasible research method for diagnosing and evaluating the treatment effects of disease microvascular function.

### Electronic supplementary material

Below is the link to the electronic supplementary material.

Supplementary Material 1

## Data Availability

The datasets used and analysed during the current study are available from the corresponding author on reasonable request.

## Abbreviations

CMR: cardiac magnetic resonance
DM: diabetes mellitus
EDV: end-diastolic volume
EF: ejection fraction
ESV: end-systolic volume
FBG: fasting blood glucose
GPS-L: longitudinal global peak strain
HDL: high density lipoprotein cholesterol
LDL: low-density lipoprotein cholesterol
MaxSI: maximal signal intensity
LV: left ventricular
MPRI: myocardial perfusion reserve index
MPR: myocardial perfusion reserve
PI: perfusion index
TC: triglyceride
