# Supplementary material for: Early left ventricular microvascular dysfunction in diabetic pigs: a longitudinal quantitative myocardial perfusion CMR study
Source: Cardiovasc Diabetol. 2024 Jan 6;23:9. doi: 10.1186/s12933-023-02106-w (PMC10771679; doi:10.1186/s12933-023-02106-w)
Supplement: Supplementary file 1 — Supplementary Material 1 [file 12933_2023_2106_MOESM1_ESM.docx]

**﻿Table S1. Reproducibility of myocardial perfusion measurement.**

|  | **﻿Intra-observer** | | **﻿** | **Inter-observer** | |
| --- | --- | --- | --- | --- | --- |
|  | **﻿ICC** | **﻿95% CI** |  | **﻿ICC** | **﻿95% CI** |
| Upslope at rest | 0.981 | 0.926-0.990 |  | 0.955 | 0.901-0.988 |
| ﻿Upslope at stress | 0.978 | 0.947-0.989 |  | 0.946 | 0.897-0.979 |
| MaxSI at rest | 0.989 | 0.961-0.995 |  | 0.972 | 0.935-0.986 |
| MaxSI at stress | 0.982 | 0.915-0.991 |  | 0.964 | 0.923-0.985 |
| MPRI | 0.929 | 0.891-0.964 |  | 0.924 | 0.901-0.983 |
| MBF at rest (mL/g/min) | 0.976 | 0.933-0.991 |  | 0.953 | 0.875-0.983 |
| MBF at stress (mL/g/min) | 0.987 | 0.965-0.995 |  | 0.975 | 0.932-0.991 |
| MPR | 0.961 | 0.893-0.986 |  | 0.925 | 0.806-0.972 |

MaxSI, maximal signal intensity; PI, perfusion index, MPRI, myocardial perfusion reserve index; MBF, myocardial blood flow; MPR, myocardial perfusion reserve.
